# *GCM2* p.Tyr394Ser variant in Ashkenazi Israeli patients with suspected familial isolated hyperparathyroidism

**DOI:** 10.3389/fendo.2023.1254156

**Published:** 2023-12-07

**Authors:** Auryan Szalat, Shoshana Shpitzen, Rena Pollack, Haggi Mazeh, Ronen Durst, Vardiella Meiner

**Affiliations:** ^1^ Endocrinology and Metabolism Service, Department of Internal Medicine, Osteoporosis Center, Hadassah-Hebrew University Medical Center, Jerusalem, Israel; ^2^ Center for Research, Prevention and Treatment of Atherosclerosis, Hadassah-Hebrew University Medical Center, Jerusalem, Israel; ^3^ Department of Surgery, Hadassah-Hebrew University Medical Center, Jerusalem, Israel; ^4^ Department of Genetics, Hadassah-Hebrew University Medical Center, Jerusalem, Israel

**Keywords:** primary hyperparathyroidism, *gcm2*, familial hypocalciuric hypercalcemia, calcium-sensing receptor, *AP2S1*, parathyroid, hypercalcemia

## Abstract

**Context:**

A germline mutation can be identified in up to 10% of patients with primary hyperparathyroidism (PHPT). In 2017, a high frequency of the *GCM2* [(NM_ 004752.4) c.1181A> C; p.Tyr394Ser; rs142287570] variant was reported in PHPT Ashkenazi Jews (AJ).

**Objective:**

To evaluate the presence of the *GCM2* p.Tyr394Ser variant in Israeli patients addressed for genetic evaluation to characterize their phenotype and clinical management.

**Method:**

Patients with PHPT who underwent addressed for genetic screening for suspected familial hypocalciuric hypercalcemia (FHH), a family history of isolated hyperparathyroidism (FIHP), or failed parathyroidectomy with persistent PHPT were recruited. Those with normal initial selected gene sequencing or hyperparathyroid genetic panel completed the *GCM2* p.Tyr394Ser variant sequencing. The prevalence of this variant was evaluated using our local genomic database.

**Results:**

A total of 42 single individuals from unrelated kindreds were evaluated. A disease-causing mutation was found in 11 (26.1%) patients: 10 were diagnosed with FHH (eight *CASR* and two *AP2S1* mutations), and one patient had a CKN2B mutation. In 28 of the remaining patients, the *GCM2* p.Tyr394Ser variant was positive in three (10.7%), and all were AJ. Within AJ (15/28, 53.5%), the rate of the p.Tyr394Ser variant was 3/15 (20%), and of those, two had a history of familial isolated hyperparathyroidism. Multi-glandular parathyroid adenoma/hyperplasia was also observed in two of these patients. No clinical or laboratory findings could discriminate patients with the *GCM2* p.Tyr394Ser variant from those with FHH. Cinacalcet normalized the calcium levels in one patient. The prevalence of the *GCM2* p.Tyr394Ser variant in 15,407 tests in our local genomic database was 0.98%.

**Conclusion:**

In contrast to previous observations, the *GCM2* p.Tyr394Ser variant-associated phenotype may be mild in AJ with FIHP, sometimes mimicking FHH. Because surgery may be curative, surgeons should be aware of the possibility of multiple gland diseases in these patients. The clinical spectrum and clinical utility of screening for this variant warrant further investigation.

## Introduction

Primary hyperparathyroidism (PHPT) is a frequently encountered endocrine disorder with an increasing incidence worldwide (1, 2). PHPT is usually diagnosed by routine laboratory tests in asymptomatic patients or patients with normocalcemic PHPT (2, 3). The disease results from a single parathyroid adenoma (PTA) in 80% of cases and from multiple glandular adenomas or hyperplasia (MGH) in 20% of cases; parathyroid carcinoma (PTC) is rare, with an incidence of less than 1% (4). Surgery is the only curative treatment (2, 5, 6). Germline mutations may be identified in up to 10% of cases (3), warranting genetic evaluation in young patients below 30 years of age with PHPT, or patients with a familial history of isolated PHPT (FIHP), or presenting clinical and/or laboratory features suggesting a syndromic disease (2): familial hypocalciuric hypercalcemia (FHH) subtypes due to mutation in the calcium-sensing receptor (*CASR*), the guanine nucleotide-binding protein subunit alpha11 (*GNA11*) or the adaptor protein complex-2 subunit sigma (*AP2S1*), or tumoral syndromes such as Multiple Endocrine Neoplasia (MEN) type 1, 2A, and 4, PHPT-Jaw Tumor Syndrome associated with various genetic mutations.

Most cases of FIHP have no genetic diagnosis, but in some cases, variants in the *GCM2* encoding for the chorion-specific transcription factor GCM2 protein may be found (3, 7), although their penetrance and pathogenetic significance remain unclear (3, 8, 9). The *GCM2* gene (OMIM #603716) localizes to 6p24.2, encodes a key transcription factor for 506 amino acids in parathyroid embryogenesis and development, which remains expressed at the adult stage (10). It has been shown that *gcm2*-null mice develop parathyroid hypoplasia and hypoparathyroidism (11), and that homozygous and heterozygous germline inactivating *GCM2* pathogenic variants in humans lead to hypoparathyroidism (12, 13). Other variants are associated with hyperparathyroidism (14, 15), with an overall higher frequency of GCM2 variants in sporadic and familial PHPT than in the general population (8, 16). In 2017, Guan et al. (17) reported that the *GCM2* p.Tyr394Ser rs142287570 variant was found in 41% and 27% of Ashkenazi Jews (AJ) with FIHP kindreds and sporadic PHPT in their referral cohort, respectively, and at a lower prevalence in other European origin individuals.

The aim of the present study was to evaluate the presence of the *GCM2* p.Tyr394Ser variant in Israeli patients being evaluated for potential or definite familial PHPT, and to describe the clinical and laboratory presentation as well as the management of patients with the *GCM2* p.Tyr394Ser variant.

## Patients and methods

Patients diagnosed with PHPT with no clinical or laboratory findings suggestive of MEN1, MEN2A, or Jaw-Tumor Syndrome were referred to our specialized center for genetic evaluation. The inclusion criteria for genetic study enrollment were suspected FHH based on low urinary fraction excretion of calcium in the context of elevated blood calcium and/or PTH levels, with or without familial history of hyperparathyroidism, patients with a diagnosis of familial PHPT based on biochemical tests and a first-degree familial history of hyperparathyroidism, young age (<30 years old), and failed parathyroidectomy with persistent disease.

Following informed consent, DNA was extracted from the leukocytes and stored for future evaluation. Until 2017, Sanger sequencing using adapted primers was performed first only for *CASR* (NM_000388.4) (18) and then completed after 2013 (19, 20) for the hotspot *AP2S1* pathogenic rs397514499 variant (p.Arg15Leu) and *GNA11* (NM_002067.5), as previously described (21). In this cohort of patients with an initial normal genetic screening, retrospective focused analysis of the *GCM2* (NM_004752.4) p.Tyr394Ser was performed by Sanger sequencing. Patients addressed for molecular studies since 2018 were evaluated using a genetic panel for PHPT, including *CASR, AP2S1*, *GNA11*, *CDC73*, *MEN1*, *CDKN1A*, *CDKN1B*, *CDKN2B*, *CDKN2C*, and *RET*. The test was performed using the xGen Exome Research Panel IDT-V2 kit in combination with xGen Human mtDNA Research Panel v1.0 on NovaSeq 6000. Fast Q data were computer-analyzed. The changes were compared to those in the human genome (hg19). Common changes (MAF >1% in gnomAD) were removed, as were non-coding synonymous changes (except for intronic changes that were less than eight bases away from a splice site) or synonymous changes that were more than three bases distant from splice sites. Quantitative change analysis was performed using the CNV-finder (software written in-house) and CNV KIT software (PMID: 27100738).

A specific review of the *GCM2* p.Tyr394Ser variant in the PHPT genetic panel was performed in patients addressed for evaluation since 2018. The presence of the *GCM2* p.Tyr394Ser variant was assessed using our local genome database, which included all genome tests performed at our facility for any medical reason. Ethnicity was assessed using a questionnaire and/or anamnesic assessment. Specifically, Ashkenazi origin was self-reported.

## Results

**Figure 1:**
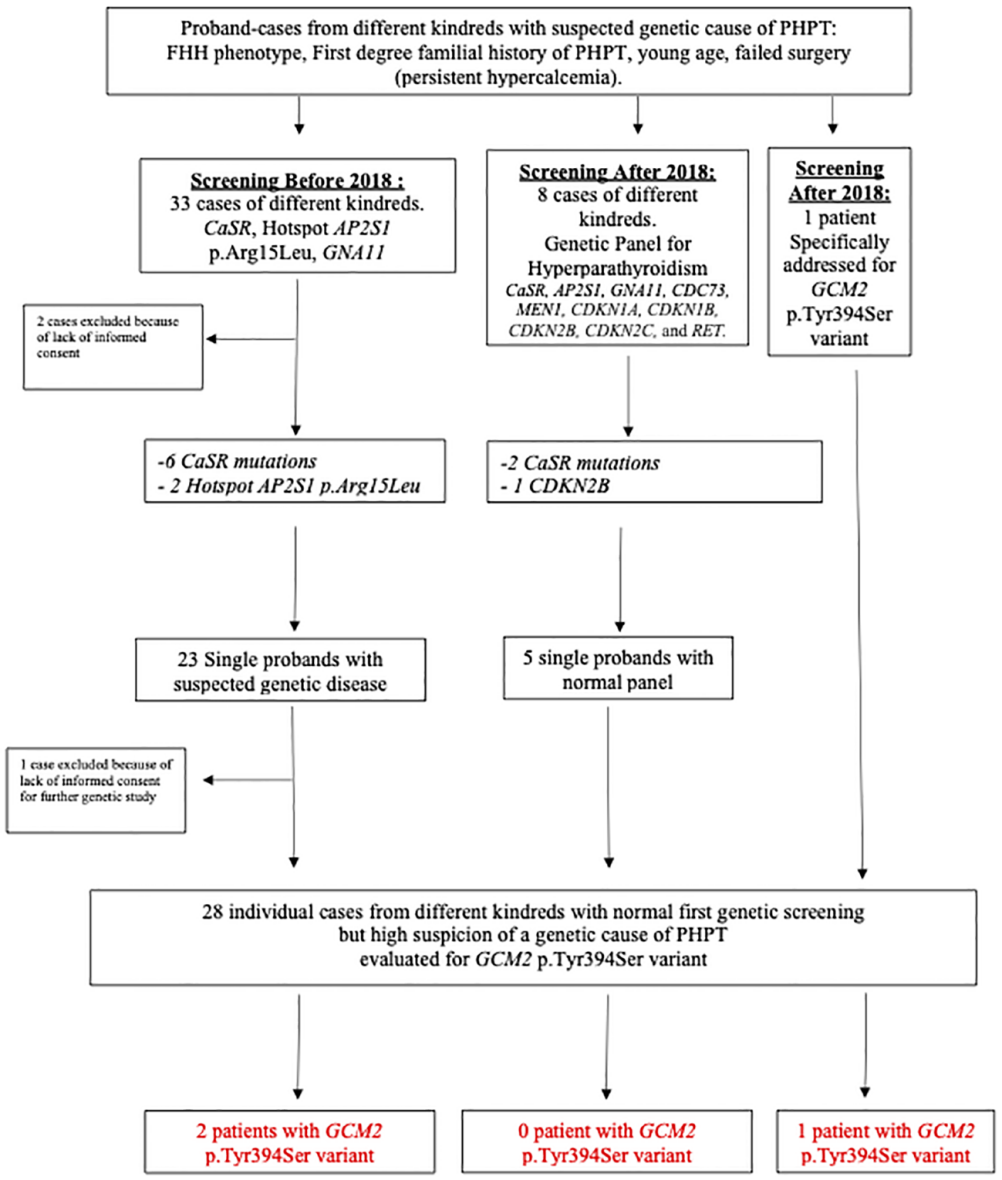
Chart of patients’ selection and results. CASR, calcium-sensing receptor; FHH, familial hypocalciuric hypercalcemia; PHPT, primary hyperparathyroidism.

Overall, 42 probands from distinct kindreds were addressed for genetic evaluation of PHPT at our center since 2009 (**Figure 1**). Two patients had no genetic evaluation because informed consent was not obtained. A total of 31 patients underwent an evaluation before and eight after 2018, respectively, and one patient was specifically addressed to test the *GCM2* p.Tyr394Ser variant as she was of Ashkenazi origin with FIHP without any syndromic feature (see **Figure 1** describing the chart of patients’ selection).

We identified 11 families (11/42, 26.2%) with a relevant genetic variant (**Figure 1**): one with a *CDKN2B* mutation, two with hotspot *AP2S1* p.Arg15Leu, and eight with *CASR* mutations.

In those evaluated before 2018, 23 patients from different kindreds remained without a genetic diagnosis, but one patient did not sign an informed consent form to conserve the DNA sample to perform additional specific genetic tests related to hyperparathyroidism. Sanger sequencing of the *GCM2* p.Tyr394Ser variant was performed on 22 patients who provided informed consent.

Among the eight patients addressed since the beginning of 2018 who had an appropriate genetic panel for PHPT, three had a positive genetic variant (**Figure 1**). In the remaining five patients, an analysis of the *GCM2* p.Tyr394Ser variant was performed. Overall, 28 Israeli patients with FIHP, young age, or failed surgery underwent a targeted evaluation of the *GCM2* p.Tyr394Ser variant (**Figure 1**). Of these, 15/28 (53.5%) or 25/42 (59.5%) of the full original cohort comprised of different kindreds, were from Ashkenazi origin, while 13/28 (46.4%) and 17/42 (40.5%) of the full cohort were from non-Ashkenazi backgrounds. Among the tested individuals, 3/28 (10.7%) patients, or 3/42 (7.1%) of the fully tested and untested cohorts, were found to be heterozygous for the *GCM2* p.Tyr394Ser variant. Overall, among the 15 Ashkenazi patients with clinical reasons to diagnose or suspect a familial state of PHPT and who agreed to our testing protocol, the prevalence of the *GCM2* p.Tyr394Ser variant was 3/15 (20%), which represented 3/25 (12%) of all the patients from Ashkenazi origin, two of which were identified within the context of the retrospective analysis, and one individual was enrolled specifically for screening for the *GCM2* p.Tyr394Ser (**Figure 1**, **Table 1**, Cases 1–3). Four other patients who were identified with FHH and a disease-causing pathogenic variant in *CASR* or *AP2S1* were also tested for the *GCM2* p.Tyr394Ser variant to identify a possible double hit. This was not observed in any of these patients (**Table 1**).

**Table 1:** Features of patients with isolated primary hyperparathyroidism diagnosed with a *GCM2* p.Y394S variant, *CASR*, and *AP2S1* mutations.

	Sex/ Age	B. Ca	U.FeCa (%)	PTH	25OHD	Imaging Study	Added Data	Surgery–Pathology
*GCM2* Y394S								
Patient 1	M/66	10.8	0.9	5.7 pmol/l (NR 1.6–6.9)	29	NA	Pseudo-gout, Osteoporosis	No Surgery
Patient 2	M/19	12	0.9	110 pg/ml (NR 15–65)	26	Neg. U/S, MIBI, CT4D.	Celiac	Failed surgery—resection of 1 PTA. Persistence.
Patient 3	F/71	10.6	1.7	46 pg/ml (NR 12–36)	32.6	LLL PTA on U/S. MIBI, LLL + RU PTA	Osteopenia MNG	Successful 3.5 glands PTX. Hyperplasia.
*CASR*								
153-001	F/14	11.3	0.7	103 pg/ml (NR 15–65)	20	Neg.		Failed Explorative neck surgery.
216-001	F/31	11.8	1.3	60 pg/ml (NR 15–65)	25	Neg.		3.5 glands resection. Hyperplasia.
224-001	F/71	10.6	1.5	35 pg/ml (NR 6–36)	28	NA	Osteoporosis	No Surgery
*AP2S1*								
151-001	F/5	12.9	0.2	60 (NR 15–65)	18.5	Neg.	Cognitive disorder. Depression. MVP.	No Surgery

Ca, calcium; MIBI, sestamibi, scintigraphy; MNG, multinodular goiter. MVP, mitral valve prolapse; NA, not available; Neg., negative; NR, normal range; PTA, parathyroid adenoma; PTX, parathyroidectomy; Susp., suspected; U.FeCa, urinary fraction excretion of calcium (calcium-to-creatinine clearance ratio): (U.Ca X P. Cr)/(P. Ca X U. Cr); U/S, ultrasound; 4DCT: 4-dimensional neck computerized tomography; 25OHD, 25-hydroxyvitamin-D levels.

Normal ranges: Blood calcium, 8.4 mg/dL–10.5 mg/dL; blood magnesium: 0.7 mmol/L–0.95 mmol/L; PTH, the normal range varied from one used kit to the other; 25-OH-vitamin D, 20 ng/mL**–**50 ng/mL.

### Case 1

The first patient was a 66-year-old man referred for evaluation in 2010. He was diagnosed with chondrocalcinosis and his sister was diagnosed with PHPT. She underwent successful surgery with resection of one PTA. Routine blood tests revealed abnormally non-suppressed PTH levels with elevated calcium levels. The patient declined to undergo surgery and was successfully treated with low-dose cinacalcet. The genetic evaluation was initially negative for mutations in *CASR*. In 2013, after mutations in *AP2S1* and *GNA11* were reported, genetic screening for these two genes was performed and the results were normal. In the present case, a retrospective analysis of the *GCM2* p.Tyr394Ser variant, which was positive, was performed.

### Case 2

The second patient was examined in 2016. The patient was a 19-year-old male, with a recent diagnosis of celiac disease. Biochemical tests revealed PHPT. Complete imaging work-up was negative (ultrasound, sestamibi parathyroid scan, 4-dimensional neck computerized tomography). Explorative surgery revealed only one enlarged PTA that was resected. On pathology, the gland was 0.5 cm and weighed 196 mg. Postoperatively, PTH levels decreased from 146 to 81 pg/ml (normal range: 15–65 pg/ml). Calcium remained elevated 10.9 mg/dl (normal range:8.4–10.5). No family history of PHPT was obtained, yet only the mother and one brother performed blood tests, which were normal, whereas the father and four other brothers and sisters did not undergo familial laboratory screening. The patient was referred for genetic evaluation because of failed parathyroidectomy (persistent hypercalcemia). Initially, no mutation was found in the *CASR*, hotspot *AP2S1* p.Arg15Leu, or *GNA11* gene. In this patient the *GCM2* p.Tyr394Ser variant was retrospectively evaluated for the purpose of this study and was found positive. As he had persistent PHPT, multiglandular disease was suspected, but he declined a second surgery and was lost to follow-up. Familial hyperparathyroidism could not be ruled out because of an incomplete familial evaluation.

### Case 3

The third patient was a 71-year-old woman with a familial history of PHPT in her mother and sister, who were cured following parathyroidectomy (two resected PTA in her sister and one PTA in her mother). She was asymptomatic and underwent blood tests compatible with PHPT. Imaging studies were suggestive of more than one PTA, and she underwent explorative neck surgery, which revealed four-gland hyperplasia with a satisfying decrease in PTH only after the third gland was resected. After 3 and one-half parathyroid gland resection surgery, calcium and PTH levels normalized and remained normal over 2 years of follow-up. Pathology revealed parathyroid hyperplasia with significant increased weight and size of the resected parathyroid glands of 580 mg/1.6 cm, 550 mg/1.5 cm, 330 mg/0.9 cm and the fourth half one being 100 mg/0.5 cm.

Among the three Ashkenazi patients harboring the *GCM2* p.Tyr394Ser variant, two were categorized as FIHP. A doubt persisted for the third patient (Case 2) because no complete familial evaluation was performed. Multiglandular parathyroid hyperplasia)MGH) was found in two patients (suspected in Case 2, proven in Case 3), whereas again, doubt remains concerning the third patient (Case 1) who did not undergo surgery. A review of the clinical, laboratory, imaging, and pathologic findings (**Table 1**) in our patients with the *GCM2* p.Tyr394Ser variant showed that all patients had asymptomatic disease and mild (patients 1 and 3) or moderate (patient 2) hypercalcemia (**Table 1**), with mild to moderate elevated PTH. Most of the patients had no osteoporosis. No discriminative parameters distinguished them from patients with FHH (*CASR* or *AP2S1* mutations). In both groups of patients, the urinary fraction excretion of calcium (uFECa) was closely below 1% or up to 2%, the serum PTH and calcium levels were similarly elevated. uFECa was very low only in only one patient with an *AP2S1* p.Arg15Leu hotspot mutation (patient 151-001, **Table 1**).

To evaluate the overall frequency of the *GCM2* p.Tyr394Ser variant in a larger Israeli population, we checked its presence among 15,407 whole exome sequencing tests performed at the Hadassah Hebrew University Hospital, and identified 141 heterozygous positive carriers of the p.Tyr394Ser variant (0.9%). None of the patients had hypercalcemia or hyperparathyroidism.

## Discussion

The phenotype of our patients with the *GCM2* p.Tyr394Ser variant presented with asymptomatic mild to moderate hypercalcemia, and are low urinary calcium excretion fraction was below 1% or below 2% and may thus overlap with the phenotype observed in FHH. However, in these cases, a classic mutation in *CASR* should be suspected first, as these mutations seem more frequent than *GCM2* variants in this context. Indeed, in our cohort, based on the same inclusion criteria for genetic screening as utilized above, we found most frequently a *CASR* variant: 8/42 (19%) index-cases from non-related kindreds (**Figure 1**), whereas we found *GCM2* p.Tyr394Ser variant in 3/42 (7.1%) and hotspot *AP2S1* p.Arg15Leu variant in 2/42 (4.7%) addressed patients. We also found one case of *CDKN2B* mutation (1/42, 2.3%). We found no case of the *GNA11* variant, which is known to be rare (22). We also observed that more than one PTA or glandular hyperplasia was found in patients with the *GCM2* p.Tyr394Ser variant. However, in contrast to cases with FHH due to pathogenic variants in *CASR*, *AP2S1*, or *GNA11*, surgery may cure the disease (Case 3) unless hyperplastic glands or other parathyroid adenomas are missed during the first surgery in cases of MGH (Case 2). Our clinical findings expand and tend to contrast with a message from previously published descriptions of more severe disease associated with *GCM2* variants; for example, a previous genotype–phenotype correlation study in 18 patients with *GCM2* variants compared to 457 patients with sporadic PHPT suggested higher preoperative levels of PTH, higher rate of multiglandular parathyroid disease (78% versus 14.3%), a lower cure rate (86% versus 99%), and a higher risk of PTC (5% versus 0%) (23) in patients with *GCM2* variants; another study described rare cases of PTC in the context of *GCM2* variants (24). Finally, patients with *GCM2* variants and FIHP as compared to ones without *GCM2* variants had significantly larger resected parathyroid glands (15). We showed that hypercalcemia could be successfully controlled with cinacalcet in one patient (Case 1) with the *GCM2* p.Tyr394Ser rs142287570 variant, and that resection of more than one gland may be necessary to cure the disease as multiple parathyroid glands can be involved, otherwise leading to failed surgery.

In this study of a highly selected cohort of Israeli patients with PHPT addressed for genetic evaluation, we found a heterozygous *GCM2* p.Tyr394Ser variant in 3/28 (10.7%) patients. This represents 3/42 (7.1%) of the full cohort with different kindreds. Among the AJ patients, 3/15 (20%) or 3/25 (12%) were included in the full cohort. Based on these findings, a recent abstract described the presence of the *GCM2* p.Tyr394Ser variant in 2/40 (5%) self-reported AJ patients with PHPT, including one familial case of PHPT, whereas it was found in 3/200 (1.5%) healthy AJ controls (25).

This shows an over-representation of the evaluated variant compared to the general Israeli population, as evaluated in the presence of the variant in our local genome database (141/15,407, 0.98%). However, this prevalence would be higher if calculated among Ashkenazi Jews tested only (approximately 1.8%). Data available from gnomAD v.4.0.0 (https://gnomad.broadinstitute.org/variant/6-10874335-T-G) show an allele frequency of the variant of 1.26% in AJ (26). These observations, especially in an Israeli population where AJ patients with FIHP can occasionally (and apparently more than in the general population) carry the *GCM2* p.Tyr394Ser variant, warrant further and larger studies to evaluate its penetrance and whether screening for its presence might have a useful role in clinical management. Moreover, the exact pathophysiology explaining how the *GCM2* p.Y394S variant acts as a proto-oncogene leading to familial PHPT needs to be further studied, as a mouse knock-in model inserting this variant using CRISPR/Cas9 gene editing technology did not reveal any serum calcium and PTH changes, nor histological, parathyroid gland size, or cell proliferation changes in genetically modified mice (27).

Our report has several limitations in addition to the selection bias in the way our patients were recruited, which focused on patients highly suspected of genetic cause for PHPT: First, it included only a small number of patients with *GCM2* p.Tyr394Ser rs142287570 variant; second, as only one specific *GCM2* variant was sequenced in 23 of the included patients (22 from a retrospective analysis of samples obtained before 2018 and one prospectively screened, **Figure 1**), other *GCM2* variants may have been missed, as well as other disease-causing mutations in other genes. Finally, the genetic evaluation we performed could not allow haplotype analysis of the patients with the p.Tyr394Ser rs142287570 to classify the variant as a founder mutation in AJ with PHPT.

In summary, in contrast with previous observations, the phenotype associated with the *GCM2* p.Tyr394Ser variant in AJ with FIHP may be mild, sometimes mimicking FHH. The clinical spectrum and clinical utility of screening for this variant warrant further investigation.

## Data availability statement

The original contributions presented in the study are included in the article/supplementary material. Further inquiries can be directed to the corresponding author.

## Ethics statement

This study involving humans were approved by Hadassah Local Institutional Review Committee. The Study was conducted in accordance with the local legislation and institutional requirements. Written Informed Consent for participation to this study was provided by all participants or by the participants' legal guardians/next of kin.

## Author contributions

AS: Conceptualization, Formal Analysis, Investigation, Methodology, Project administration, Supervision, Validation, Writing – original draft, Writing – review & editing. SS: Data curation, Investigation, Writing – review & editing. RP: Data curation, Investigation, Writing – review & editing. HM: Data curation, Investigation, Writing – review & editing. RD: Data curation, Investigation, Writing – review & editing. VM: Data curation, Validation, Writing – original draft, Writing – review & editing.
